# Exploring Influencing Factors on Help-Seeking Behaviors for Intimate Partner Violence: Insights from Ethnic Women Survivors in Myanmar

**DOI:** 10.3390/ijerph22060899

**Published:** 2025-06-05

**Authors:** Aye Myat Myat Win

**Affiliations:** Virtual Summer Research Program, Center for Research, Policy and Innovation, Burmese American Community Institute, Indianapolis, IN 46227, USA; ayemyatmyatwin23@gmail.com

**Keywords:** help-seeking behaviors, intimate partner violence, ethnic minority women, Myanmar

## Abstract

Although the help-seeking behaviors of intimate partner violence survivors have been imperative, less evidence has been shown on ethnic women’s help-seeking behaviors. As such, this study aims to explore women survivors’ help-seeking behaviors in Kachin State, an ethnic area of Myanmar with an online in-depth interview approach. Twelve women survivors who were recruited by social workers, camp leaders, and volunteers participated in the study. Findings were analyzed by using the intimate partner violence help-seeking theory. In contrast to the extant literature, this study found an equal number of women survivors reached out to both formal and informal sources. Their help-seeking behaviors were influenced by their violence perception, cultural norms, lack of information on support services availability, the violence severity, and social support. Exposure to parental violence and women’s unique personal attributes had a negative impact on their decision to seek formal help. Results underscored the importance of sociocultural norms in an ethnic patriarchal society. As such, addressing deep-rooted patriarchal sociocultural norms, effective awareness raising, women’s empowerment, establishment of anti-domestic violence law, and multi-sectoral collaboration with men’s engagement and inclusion of cultural and religious leaders are urgently needed in ethnic communities to enhance formal help-seeking.

## 1. Introduction

Intimate Partner Violence (IPV), a common form of violence against women, has been a public health and social issue that has brought attention to diverse scholars for several decades [1,2]. According to the World Health Organization [3], IPV is defined as “behavior by an intimate partner or ex-partner that causes physical, sexual or psychological harm, including physical aggression, sexual coercion, psychological abuse and controlling behaviors”. The WHO highlighted that around 30 percent of women experience some type of IPV from their intimate partner throughout their lives [3]. The occurrence of IPV varies depending on the country [4], and it is higher in developing countries [5], including those in the Southeast Asia region [3]. A study in Myanmar showed that two-thirds of the married participants have experienced physical violence; 61.5% and 13.9% of the participants have experienced sexual and emotional violence, respectively [6]. Although IPV can occur regardless of culture, economic status, and geographical area [1,7], individuals from lower socioeconomic backgrounds, with limited community support, and ethnic minorities are particularly vulnerable to encountering gender-based violence (GBV) [8,9].

Women who experience IPV face both physical and mental health consequences. Physical consequences involve injuries, sexually transmitted infections, sexual and reproductive health problems, and even suicide. Mentally, women survivors can experience fear, anxiety, depression, dependency on alcohol and drugs, and disturbance of daily routine [10,11,12,13,14,15,16]. Furthermore, IPV results in lifelong trauma such as post-traumatic stress disorder and self-harming behaviors. As a result, it has a negative impact on family, community, and society at large. Consequently, help-seeking becomes imperative to reduce the consequences of IPV and promote the general well-being of the survivors [17]. However, violence cases are remarkably underreported [18] since the survivors are hesitant to disclose their violence due to many factors, including shame and social stigma [19]. Thus, it is considered a “tip of the iceberg or silent epidemic” [20]. This situation is particularly imperative in Myanmar women who are reluctant to express their feelings and willing to maintain their family for the sake of the children [21]. Specifically, IPV help-seeking is important in Myanmar ethnic women who are confined in their cultural and marital social norms.

Survivors can approach both formal service providers, such as police, lawyers, and response organizations, and informal systems that involve family and friends for support services [22]. Nevertheless, the majority seek support mostly from informal networks such as family, relatives, friends, neighbors, and colleagues [23,24,25,26]. Women who seek help from formal institutions are scarce despite the high prevalence of violence [27,28,29,30,31], and they feel frustrated by receiving response services [32]. The survivors from developing countries rarely look for help due to limited available services or fear of re-victimization or potential danger for their family [30], and most tend to seek help from informal sources [25]. Particularly, women living in low-income countries with high gender inequality seek less help than those in low gender inequality countries [27,28].

Social and patriarchal norms that condone violence are prevalent in Myanmar culture, especially in the ethnic minorities [33]. Ethnic identity means a person’s sense of self that belongs to a specific cultural group [34,35]. Myanmar is composed of eight major ethnic groups with more than a hundred ethnic sub-groups. Domestic violence exists as a hidden problem [36]. Women survivors keep their abuse quiet, and conducting violence research on ethnic minority women is suggested [37].

Studies of racial minority women have shown that they are less likely to seek support from social services [38,39]. Religion and cultural beliefs, value of family honor, and community influences to remain in the violent relationship are barriers for women survivors’ formal help-seeking [40,41]. Patriarchal cultural norms induce women’s responsibility to maintain their marriage, which further hinders their help-seeking. The cultural acceptance of violence also prevents women’s IPV recognition [41]. Black women avoid outside interference for IPV due to shame, pride, consideration as a private matter, and the strong black women stereotype. Adherence to Christian religious teaching and beliefs causes ethnic women to cope with their IPV experiences [38,41]. Moreover, lack of desire to seek help, perception of the violence as minor, institutional racism, financial dependency on the husband, and lack of knowledge on available support services are revealed as reasons for not seeking help from social services [17,39,41].

On the other hand, IPV severity, polyvictimization, fear that lives are in danger, old age and low income, and concern for children’s safety lead women to seek formal help [17,39,40]. However, having children can also result in not seeking formal help due to a hesitancy to leave their husbands despite their desire to protect their children [17,42]. Regarding IPV types, women experiencing sexual violence and stalking are more likely to seek formal help than women with psychological violence experiences [42].

Ethnicity affects the types of formal and informal sources for help-seeking. The perception of keeping family problems private leads to reaching out to informal sources of support. Furthermore, socioeconomic differences play a role in the differences in ethnic women’s help-seeking behaviors. Women with high socioeconomic status are reluctant to view their experiences as violence, and their dissatisfaction in dealing with police results in not seeking formal help. In addition, lack of and/or perceived lack of culturally aligned services, geographic and financial barriers, and distrust in health care personnel lead ethnic women to seek help from informal sources [42].

Much evidence has indicated the importance of help-seeking in IPV survivors. A recent study mentioned poor help-seeking as a remarkable public health problem [28]. A number of studies proposed to explore more about help-seeking experiences of the survivors [22,24], including in racial/ethnic groups [43]. Studies on IPV help-seeking behaviors emphasize rural and urban women [44], and migrant and immigrant women [45,46,47], with limited emphasis on ethnic women.

Consequently, this study aims to analyze help-seeking behaviors of intimate partner violence women survivors in an ethnic minority area. This is the first study that explores IPV help-seeking behaviors among ethnic women survivors in Myanmar, and specifically in Kachin State, one of the ethnic areas in the country. Ethnic women living in Kachin State are the study participants. Extant studies in Kachin State emphasize children’s mental health and psychosocial support, and risk factors of domestic violence [38,39]. As such, evidence on IPV help-seeking behaviors in the area has been overlooked. Hence, this study will address the gap in the literature with the aim of exploring the help-seeking behaviors of IPV women survivors in Kachin State, Myanmar, and the factors influencing their help-seeking behaviors.

Various theories have been utilized to understand IPV women survivors’ help-seeking behaviors. Firstly, based on McLeroy et al. [48] social–ecological model, individual characteristics and interpersonal, community, and system factors affect women survivors’ IPV disclosure [49]. Secondly, the survivor theory views women as empowered survivors, not as victims in help-seeking [50]. As such, women seek formal help in experiencing severe violence from their husbands [42]. Thirdly, Bandura’s social cognitive theory emphasizes self-efficacy [51], underscoring the survivors’ agency. Fourthly, Crenshaw’s intersectional theory highlights the intersection of various social categories such as gender, race, class, etc. [52], and their combination and reinforcing effects in women’s IPV experiences and responses, including help-seeking behaviors [53]. Lastly, Liang et al. [54] help-seeking framework mentions three stages of seeking help: “defining the problem, deciding to seek help, and selecting a source of support”. In the first stage, the survivor evaluates and defines the problem. If she does not perceive the problem as violence, she will not enter the next stage. Therefore, proceeding to the second stage is based on her perception of the problem. In the second stage, the survivor usually decides to seek help when the violence is intolerable and realizes that the problem will not go away without others’ help. In the final stage, the survivor will choose the type of support she will seek, such as formal and informal sources. However, the stages may not always be a linear process, but an interactive one. In addition, multiple factors at individual, interpersonal, and sociocultural levels influence each stage of help-seeking [54]. The study utilized Liang’s help-seeking framework to explore the diverse influencing factors at different stages of the help-seeking process.

### Study Context

Myanmar, a country in the Southeast Asia region, is composed of various ethnic groups and diverse cultures. A patriarchal gender structure is prominent in the country from the precolonial, colonial, to the current military rule, with men holding authority in every area, such as political, economic, and social spheres. Men’s superiority is culturally denoted by “hpon” (power, glory) that triggers women’s inferiority [37,55]. Men are assumed as breadwinners, whereas women are responsible for housework within the family [56,57]. Moreover, the country has suffered civil conflicts for many decades, which has had a negative impact on its economic and educational development [58,59]. Hence, political instability and women’s socioeconomic marginalization are linked with IPV [60,61,62]. Despite the ratification of the Convention on the Elimination of All Forms of Discrimination Against Women (CEDAW) in Myanmar [63], women still encounter high IPV risk [64].

Social inequality is especially prominent in Myanmar’s ethnic areas, including Kachin State, which is located in the northern part of the country. Kachin culture is tremendously patriarchal, with a low status of women in economic, social, cultural, and political areas. Even though there are some changes in modern times, conservative norms continue to dominate in the area. Traditional gender roles are maintained within the family and reinforced outside the household, such as doing housework and childcare are women’s tasks, while the husband is the head of the family and breadwinner. In this way, conservative sociocultural norms have a significant influence on society [65]. Furthermore, the civil conflict exacerbated after the coup d’état in 2021, which negatively affected livelihoods and gender roles, resulting in women’s IPV experiences [37,60,66,67]. IPV cases have dramatically risen, with an increased number of women survivors accessing helplines. Justice for GBV survivors is a struggle in Myanmar, and it is becoming worse in the current post-coup period. Local women’s networks and civil society organizations offer support to IPV survivors that involves basic needs assistance, financial aid, safehouses, medical treatments, psychological counseling, and legal support. However, they also encounter challenges with decreased capacity and increased demand for services. Internet and telecommunication restrictions also decrease the organizations’ ability in GBV awareness raising, response, and follow-up services [68].

IPV is a major social and public health problem in Myanmar. A study in Myanmar showed that the prevalence is higher in women with low educational levels, high deci-sion-making power, and living in rural areas. Specifically, women with high economic situations are more likely to encounter sexual violence than those with low incomes [6]. Gender role beliefs of male domination and female subordination, and the violence acceptance social norms justify IPV and minimize its consequences [69].

A past study in Myanmar that analyzed the national demographic and health survey data revealed that 30% of women seek help, and the remaining 70% do not seek any form of help. The help-seeking rate is similar in women with physical and sexual violence experiences, while the rate is slightly lower in those with emotional violence experiences. Their help-seeking behaviors are positively associated with age, educational level, emotional violence experience, and living in areas such as Yangon, Mandalay, and Kayin States. On the other hand, women in high economic conditions and living in rural areas are less likely to seek help [21].

Despite extant evidence on ethnic women, mainly in the Global North, this study emphasizes ethnic women’s IPV help-seeking behaviors not only in an overlooked country in the Global South, Myanmar, but also in a patriarchal society. The study will highlight how IPV women survivors’ help-seeking behaviors in Myanmar’s ethnic areas are different from those in other nations. The findings are expected to contribute to the existing IPV help-seeking literature and have important practical and policy implications.

## 2. Method

In-depth interviews were conducted with 12 women who have experienced intimate partner violence in Kachin State, an ethnic area of Myanmar. The participants were recruited with purposive sampling with the support of social workers, including an internal displacement camp leader and volunteers. The recruitment criteria were (1) age between 18 and 64 years old and (2) experiences of physical or sexual, or psychological violence from their husbands or ex-husbands.

In the preparation phase, social workers were contacted and informed about the study and their support was requested for the recruitment of participants. The recruitment criteria were shared with them, with emphasis on voluntary participation. Social workers also shared the contact information of the internal displacement camp leader and volunteers with the author. The camp leader and volunteers also knew the women survivors living in the community. Therefore, they were also contacted and involved in the participants’ recruitment. The participants who sought both formal and informal help were included in the study due to the recruitment of participants from various gatekeepers, such as social workers, the internal displacement camp leader, and volunteers. Regardless of women survivors’ help-seeking behaviors, they were recruited based on their voluntary willingness to participate in the study.

Online interviews were conducted via the Zoom platform in a safe private room, which was arranged by the gatekeepers, and a semi-structured interview guide was used. A social worker was available near the interview location to provide support if needed. The study was explained to the participants, which included background, purpose of the study, strict confidentiality of the participants’ identity and their provided information, and their ability to withdraw from the participation at any time. After that, informed consent was received from each participant for the participation. Ethical approval was given by the human research ethics committee of the author’s former university. Interviews strictly followed the gender-based guiding principles, such as confidentiality, safety, respect, and non-discrimination, and the WHO guidelines for ethical and safety recommendations [70,71]. The interviews were conducted between February 2023 and October 2023, and they were audio- or video-recorded based on the agreement of each participant. Note taking was also conducted after obtaining permission from the participants.

After verbatim transcription of the interviews, thematic analysis [72] was used to manually analyze the data based on the Liang et al. [54] help-seeking framework. Formal support sources involved health care centers, police, non-governmental organizations, and social services, such as women’s shelters and counselors, while family, friends, colleagues, relatives, and neighbors were included in the informal support sources [22,23,24,25,26,39]. Table 1 shows the socio-demographic characteristics of the participants.

### Sociodemographic Data

Twelve women survivors were involved in the study, and most of them are from 24 to 34 years old. One-third work as daily wage laborers and have a middle school educational level. Two-thirds of the participants have between MMK 100,000 and 300,000 as monthly family income. A participant’s household does not have income because of the recent loss of jobs in both married couples. Three participants in the study are unemployed, and hence they do not have income. The majority are Baptist and are from the Jingpo and Rawang ethnic groups. For the living locations, half of the total participants live in WaingMaw Township, and one-third live in Myitkyina and MoeMauk townships, respectively. More than two-thirds of the participants have 1 to 3 children.

## 3. Results

Half of the participants sought help from formal support organizations, while the remaining half sought support from informal sources such as friends and relatives. A theoretical framework proposed by Liang et al. [54] was used to understand the help-seeking process among the IPV ethnic women survivors. The framework involves three stages: defining the problem, deciding to seek help, and selecting a source of support, which is further divided into informal and formal help-seeking. In each stage of help-seeking, diverse influencing factors at individual, interpersonal, and sociocultural levels were analyzed based on the interview data.

### 3.1. Defining the Problem

#### Individual Factor—Consideration as a Private Matter

In a society with patriarchal cultural and sociocultural norms, women do not have IPV knowledge and thus do not perceive their abuse experiences as IPV. Instead, they assume it is a private matter that hinders their support-seeking.

*“I should not tell private matters to other people and if they know a lot, it is not good. I will get a low impression if I tell my experiences in detail.”*

(Participant D, age 24)

Women’s perception of the violence as a private matter is also linked with shame to seek help. They suppose that not only should IPV not be shared with others, but also there would be no change from disclosure and help-seeking. Consequently, they keep their experiences to themselves.

*“It is my own matter. If I tell others, it doesn’t make any difference. It is even shameful. So, I don’t tell and keep it secretly, most of the time.”*

(Participant G, age 50)

### 3.2. Deciding to Seek Help

#### 3.2.1. Individual Factors

##### Priority to Housework and Childcare

Traditional gender role ideology induces men’s role as breadwinners and women as being responsible for the private sphere. As such, women are busy with housework from dawn to dark, resulting in them not seeking formal support. A participant expressed how she was occupied with household responsibilities.

*“I haven’t involved in seeking support yet since the situation does not allow it. I am busy with childcare and housework all day.”*

(Participant A, age 35)

##### Fear of Blaming and Stigmatization

Consideration of IPV as a private matter led to not disclosing and seeking support in women survivors. This consideration was also accompanied by their concern for blaming and stigmatization, resulting in a negative impact on women’s decision to seek support.

*“If I disclose to others about me, it is sure that they will look down on me and blame me instead of providing services. So, I do not tell anyone and only today, I disclose. I am afraid that others will know that I am suffering from the violence at home. When they know, I don’t want them to blame me rather than offering support services. I don’t want to take out the inside matter to outside. If I take my matter outside, I perceive that they will blame me. So, I don’t disclose it to anyone.”*

(Participant B, age 54)

Being old was found as a factor that hindered formal support-seeking. A participant expressed how her fear of stigmatization due to her age led her not to seek help. It underscores how old women have a double concern for help-seeking in a patriarchal society.

*“I have concern that they will tell me you are crazy… blah… blah… So, I just keep it to myself. I think so in my mind. They don’t tell me, but I perceive and think like that. Since I am old enough, I don’t want to tell anyone.”*

(Participant G, age 50)

In addition to patriarchal culture and subsequent perception of IPV as a private matter, concern about stigmatization stems from personal characteristics such as occupation. A woman who worked as a sex worker stated how her sex work also added to her fear of stigmatization.

*“…It is not because of others… I feel ashamed… Others will laugh at us…”Her husband beats her from argument. It is good,” like that… I don’t want others to feel happy… So, I don’t go anywhere. It is because I have faced it. And, my husband doesn’t say anything even if others tell me “prostitute”.*

(Participant L, age 31)

##### Severity of the Violence

The violence severity also played a role in women’s decisions in seeking support. A participant reported that she disclosed the violence to her friend, not to cultural and religious leaders, since her violence was not severe.

*“I don’t tell cultural and religious leaders. Since it will be a serious stage if I tell them. I also don’t want them to be busy. As you may know, cultural leaders don’t take it seriously if it is not very severe.”*

(Participant F, age 27)

In addition to the degree of violence severity, age and shame affect women’s decisions to seek support. Another participant mentioned how her age, fear of stigmatization, and minor abuse resulted in keeping her IPV experiences private.

*“For minor cases, I don’t tell others. I just endure and stay by myself. If I tell others, I am afraid that they will tell me I am crazy. So, I just keep it. Since I am old enough, I don’t want to tell.”*

(Participant G, age 50)

##### Exposure to Parental Violence

Parental violence exposure became a hindrance to women survivors’ help-seeking. A participant’s experience indicated intergenerational violence and her help-seeking decision.

*“The whole village knows that my mother was being violated by my father. But, my mother never told others. I don’t want the problem to be bigger than it needs to be. So, I feel ashamed.”*

(Participant B, age 54)

Witnessing violence both in the family and in the neighborhood normalizes IPV in women. Consequently, they perceive IPV as normal and do not proceed to help-seeking. It also underlines the importance of breaking the violence cycle in a society with a violence-acceptance attitude.

*“For these kinds of conflict, beating,* etc.*, I have seen since my childhood, “Aww… married couples are like this. It is a usual thing.” I noted in my mind like this. The most common is my parents in our ward.”*

(Participant L, age 31)

#### 3.2.2. Interpersonal Factors

##### Lack of Information on Available Services

Not knowing the available support services and organizations leads women survivors not to seek support, which underscores the imperative role of awareness raising in ethnic areas.

*“No, I don’t, even once. I don’t know and have the information about the organizations. I didn’t know it when I was living in Putao and also didn’t know how to connect with organizations when I arrived here. I also worry that other people will know about me.”*

(Participant A, age 35)

Awareness-raising sessions were provided in internally displaced camps by the community. Hence, women living in the community area did not have knowledge of support organizations, and thus could not seek help.

*“I don’t know and take services. I don’t know if there are organizations and the organizations provide support services. I don’t know it until now. Only in camp, people get training and organizations are available, But, in the community, we don’t know about that. For organizations, there is no recruitment to receive trainings, so I don’t know. I don’t receive pamphlets.”*

(Participant B, age 54)

##### Social Isolation

Being away from family, relatives, and cultural leaders was a vulnerability for a woman, which then led to her not disclosing her violent experience. Thus, social support plays an important role in women survivors’ help-seeking.

*“Since my sisters are far away, I don’t tell them. They live on the border, on the other side of Bhamo. I am here alone. I arrived here after marriage. I haven’t talked with my parents for so long due to far distance and irregular internet connection in their town… There is no phone line, only we can talk* via *WeChat. Since they don’t use WeChat, they are old enough, only younger brother uses it. As they are old enough, it has been a long time that I don’t talk with my parents. I just keep it to myself as there is no relative here and no one will stand by my side. So, I don’t tell them. My cultural leader is not here. And, since my relatives are not here, cultural leaders will not take it into account. I think like this and decided not to tell anyone and keep it to myself.”*

(Participant C, age 33)

#### 3.2.3. Sociocultural Factors

A participant emphasized the role of cultural leaders in ethnic areas. They encouraged intact marriages in addressing IPV cases. Consequently, she did not have the desire to seek support from them.

*“In the case of extra-marital affairs from husband’s or wife’s side, the cultural leaders asked whether the couple will divorce. In most cases, they don’t allow divorce and encourage reunion.”*

(Participant F, age 27)

### 3.3. Selecting a Source of Support (Informal Help-Seeking)

#### 3.3.1. Sociocultural Factors

##### Society with Violence-Acceptance Attitude

Findings showed how societal attitudes toward violence triggered women not to reach out to formal support organizations. IPV is common in a patriarchal community, and women remain in the violence cycle by emotionally supporting each other and normalizing the violence.

*“Sometimes, I tell my closest and nearest friends. They are also in the same boat. Their husbands are also like this. “Since husbands are aggressive and powerful, they do it to wives. Just like your husband does, our husbands are also the same.” Sometimes, both of them do each other.”*

(Participant C, age 33)

The violence-acceptance attitude was compounded by old age and having children. The attitude was reinforced among ethnic women survivors in their informal help-seeking.

*“I didn’t tell anyone. But, I had one friend. I frequently disclosed this to that friend. She said, “Endure. Endure, don’t tell other people. You are getting older and you have children. So, endure.” My friend told me like that.”*

(Participant G, age 50)

Women’s IPV experiences were normalized and condoned in the ethnic community, which became an obstacle for the women survivors’ formal help-seeking. Additionally, women were expected to consider children and endure in the violent relationship without seeking formal help.

*“It is not with organizations, but with friends. My friends… they told me that they also face like this and told me to endure…”Since you have many children, you continue to endure. Later, children will be good to you when they grow up.”*

(Participant E, age 28)

##### Religious Belief

Christian religious belief negatively affected women’s decision to seek help. Women were reluctant to seek help from formal support services since they wanted to maintain their marriage according to the religious perception.

*“I didn’t disclose it to religious leaders, but to my older brother. He was angry, “Can’t you divorce him? He severely bullies you,” he told me a lot. For me, I can’t do anything. Since I get married in front of God, I don’t say anything, just stay.”*

(Participant G, age 50)

### 3.4. Selecting a Source of Support (Formal Help-Seeking)

#### 3.4.1. Individual Factor—Severity of the Violence

Women reached out for formal support when they experienced severe violence in terms of frequency and severity. Additionally, knowing the availability of support organizations had a positive impact on women’s formal help-seeking. The data showed the need to emphasize IPV awareness raising in ethnic areas.

*“It is because I am afraid of him… And, I don’t want to suffer again and again… Now, since there are many NGOs, it can be said that we have open eyes and ears. Previously, I thought I should endure. But now, I have a wider perspective since there are many organizations, “Aww… we should not stay quiet. We cannot endure the whole life.” How to say… it is a kind of satisfaction, encouragement… like that…”*

(Participant K, age 33)

#### 3.4.2. Interpersonal Factor—Good Social Support

Support services information from social networks plays a vital role in help-seeking. Good social networks and family support had a positive impact on women survivors’ formal help-seeking.

*“I have talked with a staff like now as I am talking to you. It was… in 2021, my mother arrived here to avoid civil war. I told my Mom about my experiences and she talked to her friend about that. And, her friend shared the support organization information to me.”*

(Participant J, age 34)

A participant’s experience reflected the importance of public education and social support in women survivors’ formal help-seeking. Educating the public changed the women’s IPV perception, and the information was circulated within their community, which facilitated women’s utilization of formal support services for their IPV experiences.

*“I come to this staff and they encourage and support us emotionally. We are told that we can disclose our feelings. So, I come here. This organization is my first time, I have not told anyone before. It has been three to four times. I receive food and money support. They give us to the extent that they can provide even though it is not enough. I didn’t know this organization. I asked surrounding neighbors and they told me that when we are suffering too much and not ok in marriage, it is good to go to the organization. They have attended the talks and have experiences. They shared with us and connected with the organization. I also connected another woman who has difficulties in marriage to this organization. I have experienced and suffered a lot. So, if they call, I want to participate and disclose what I am suffering inside. They encourage me, no matter what happens, not to feel small, we will try. So, we want to disclose all our feelings without being ashamed.”*

(Participant I, age 48)

## 4. Discussion

The findings underscore the help-seeking behaviors and their influencing factors in an ethnic area of Myanmar. The study found an equal number of the participants reached out to both formal and informal sources, which is in contrast with extant knowledge on informal sources as a common help-seeking form both in Myanmar [21] and in other nations [30,46,73,74,75,76]. This may reflect the differences in culture and social norms among various countries. Regarding Myanmar’s findings, past study is based on the secondary data analysis of the 2015–2016 nationwide survey, while this study focuses on one ethnic area of the country recently, which limits the ability to compare the findings. While the existing literature has shown financial difficulty as a barrier to help-seeking [17,41,42], and old age leads to formal help-seeking [22,39], this study revealed old age as an obstacle for ethnic women’s decision to seek help, and the participants do not point out the impact of financial situation on help-seeking. The results show the negative impact of gender stereotypes and patriarchal norms on women survivors’ help-seeking, which is consistent with past findings [22,76].

Regarding violence recognition, the findings of this study align with past research, in which women survivors consider IPV as a family matter that will resolve itself and are not willing to disclose it to others and seek formal help [22,77]. Women who perceive IPV as a private matter were less likely to seek support than those who do not [30]. As highlighted by Liang et al. [54], the survivors’ evaluation and perception of the violence have a significant impact on help-seeking behaviors.

Many factors influence the survivors’ decision on support selection [54]. Women reported that they do not know about available IPV support services, and there is no information sharing in their areas. An extant study also revealed a lack of knowledge on available support services as one of the reasons women do not reach out to formal support organizations for their violence experiences [46]. Furthermore, they do not have time to seek formal help because of their struggle with housework. Additionally, old age negatively affects women survivors’ help-seeking decisions because of shame. Women’s exposure to parental violence also hinders their decision to seek formal support. In this way, women fall into the violence cycle in a society with patriarchal marital norms.

As help-seeking sources, women who reached out to their informal network stated how their friends and relatives faced the same, which obviously highlights ethnic women trapped in marital sociocultural norms. In Kachin society, wives are expected not to defame the honor of their clans and to respect the dignity of their spouses [65], and thus, women do not report and seek help from formal organizations for their violence. Furthermore, since women put their children as their primary concern, with a willingness to sacrifice their freedom and comfort [65], they endure in their marital relationship, as consistent with a recent study [78]. Moreover, religious belief plays a role in women survivors’ help-seeking that is also pointed out by previous studies [38,41,78]. Women survivors who disclose to their informal sources are encouraged to endure and remain in the relationships. As such, it is evident that there is a strong influence of patriarchal culture in ethnic communities.

On the other hand, they receive formal support services for psychosocial, shelter, food, and financial support only in experiencing severe violence and having good social support. This finding is supported by prior studies that showed the association between severity of violence and formal help-seeking [9,45,46,76,79,80,81]. Formal help-seeking usually comes from referral of and encouragement from family, friends, and relatives [82], as found in this study. Women know the support organizations from their family, friends, and neighbors. As a result, the violence severity and strong social network play a vital role in formal support-seeking.

### 4.1. Implications and Contributions of the Study

Addressing the barriers for ethnic women’s help-seeking is imperative to eradicate the adverse consequences of the violence [17]. Suggestions for community, system, and policy levels are provided based on the findings from this study. Results showed misconceptions and cultural normalization of the violence as alarming factors for help-seeking. As such, effective awareness raising, focusing on tackling deep-rooted patriarchal beliefs, IPV information and its consequences on women and children, the importance of help-seeking and no victim blaming, and sharing information on available formal support services are strongly suggested [38,41]. Additionally, sensitization of violence response services to community, faith-based organizations, and cultural and religious leaders is essential [40]. At the system level, support services should be qualified, confidential, and culture- and language-specific, so that ethnic women survivors can trust and rely on them [17,38,42,83]. Provision of support services should be increased to enhance accessibility, which is particularly important in the current turbulent times. Furthermore, enhancing women’s empowerment and livelihoods by IPV awareness raising, access to resources and employment opportunities, education, and skills training is crucial to increase their formal help-seeking. Lastly, at the policy level, effective anti-violence and response policies need to be well-established. The policies should emphasize the eradication of harmful gender norms and the achievement of gender equality. Most importantly, law and policy reinforcement are crucial since domestic violence law has not been ratified in Myanmar [21,37,60]. Furthermore, intimate partner violence intervention requires a multi-sector response. The coordination among various formal support service providers, such as government, education, health care, and legal support, is needed. In addition, collaboration between formal support organizations and informal sources, such as family members and various social networks, is imperative since women usually first seek support from family, friends, relatives, community, and religious leaders [17,38,42]. Communities should be educated about a survivor-centered approach and gender-based violence guiding principles to ensure quality support is delivered to the survivors.

As an empirical contribution, the study adds to the existing literature about ethnic women’s IPV help-seeking behaviors with an inclusive three-level analysis of help-seeking theory. Regarding a theoretical contribution, the study adds to the help-seeking theory from the ethnic women’s help-seeking perspective and their unique personal characteristics, such as their particular employment, age, and parental violence exposure in their help-seeking decision. Practically, the results of the study provide valuable implications to enhance community awareness and improve effective response services for the survivors.

### 4.2. Strengths and Limitations

There are certain strengths and limitations in this study. One of the strengths is the analysis of help-seeking behaviors rigorously with help-seeking theory and its influencing factors at three levels. The exploration of ethnic women’s help-seeking behaviors, and implications and contributions for this particular population, is another strength. Despite these strengths, this study focuses only on one ethnic area of Myanmar. Therefore, other ethnic areas should be targeted in future studies, and comparing help-seeking behaviors of various ethnic groups would be beneficial.

## 5. Conclusions

To the best of the author’s knowledge, this is the first study that explores help-seeking behaviors of IPV women survivors in Kachin State, Myanmar, by using the help-seeking theory and its analysis from individual, interpersonal, and sociocultural influencing factors. Misconceptions of the violence, the violence-acceptance attitudes, social norms, and parental violence exposure are mainly found as common reasons for not seeking formal support, whereas the violence severity and social support facilitate formal help-seeking. Suggestions at community, system, and policy levels are highlighted to improve formal support-seeking. To advance the knowledge on this important topic, future study should explore the perception of the survivors’ family members and cultural and religious leaders in ethnic areas toward help-seeking. Moreover, research on the effectiveness of formal response services for survivors should be conducted to gain insight and improve the quality.

## Figures and Tables

**Table 1:** Socio-demographic characteristics of participants.

Socio-Demographic Characteristics	Number (N)	Percentage (%)
Age (years)		
24–34	8	66.67
35–45	1	8.33
46–54	3	25.00
Marital status		
Married	9	75.00
Separated	2	16.67
Divorced	1	8.33
Occupation		
Unemployed	3	25.00
Daily waged job	4	33.33
Agriculture	3	25.00
Cleaner	1	8.33
Laundress	1	8.33
Household income (MMK) per month		
100,000–300,000	8	66.67
Over 300,000	3	25.00
No income but support from family and relatives	1	8.33
Educational level		
University graduate	1	8.33
High school	3	25.00
Middle school	4	33.33
Primary school	3	25.00
No schooling	1	8.33
Religion		
Baptist	10	83.33
Catholic	1	8.33
Buddhist	1	8.33
Ethnic group		
Jingpo	4	33.33
Rawang	4	33.33
Bamar	1	8.33
Shan	1	8.33
Kayin	1	8.33
Shan + Maru	1	8.33
Residence location		
Myitkyina	3	25.00
MoeMauk	3	25.00
WaingMaw	6	50.00
Number of children		
1–3	9	75.00
4–6	3	25.00

## Data Availability

Data is contained within the article.
